# Infliximab for pediatric patients with ulcerative colitis: a phase 3, open-label, uncontrolled, multicenter trial in Japan

**DOI:** 10.1186/s12887-019-1739-5

**Published:** 2019-10-13

**Authors:** Hitoshi Tajiri, Katsuhiro Arai, Seiichi Kagimoto, Reiko Kunisaki, Nobuyuki Hida, Noriko Sato, Hiroshi Yamada, Mieko Nagano, Yutaka Susuta, Kunihiko Ozaki, Kazuoki Kondo, Toshifumi Hibi

**Affiliations:** 1 Department of Pediatrics, Osaka General Medical Center, Osaka, Japan; 2 Division of Gastroenterology, National Center for Child Health and Development, Tokyo, Japan; 3 Division of General Pediatrics, Saitama Children’s Medical Center, Saitama, Japan; 4 Inflammatory Bowel Disease Center, Yokohama City University Medical Center, Yokohama, Japan; 5 Division of Internal Medicine, Department of Inflammatory Bowel Disease, Hyogo College of Medicine, Hyogo, Japan; 6 Ikuyaku Integrated Value Development Division, Mitsubishi Tanabe Pharma Corporation, Tokyo, Japan; 7 Center for Advanced IBD Research and Treatment, Kitasato University Kitasato Institute Hospital, Tokyo, Japan

**Keywords:** Infliximab, Pediatric, Ulcerative colitis, Phase 3, Multicenter trial, Japan

## Abstract

**Background:**

Pediatric ulcerative colitis (UC) is typically more extensive and has a more active disease course than adult UC, and requires early treatment augmentation to achieve and maintain disease remission. The present study aimed to investigate the efficacy, safety, and pharmacokinetic profile of infliximab (IFX) in pediatric patients with moderate-to-severe UC and inadequate response to existing treatment.

**Methods:**

This open-label, uncontrolled, multicenter, Phase 3 trial was conducted at 17 centers in Japan between April 2012 and September 2014. Pediatric patients (aged 6–17 years) diagnosed with moderate-to-severe UC received a treatment protocol comprising 5 mg/kg IFX at Weeks 0, 2, and 6, and Clinical Activity Index (CAI)-based responders at Week 8 also received treatment at 8-week intervals at Weeks 14 and 22, with a final evaluation at Week 30.

**Results:**

A total of 21 patients were treated in this study. IFX therapy rapidly improved clinical symptoms, and this effect was maintained for up to 30 weeks. Overall CAI-based remission rate was 42.9% and overall Pediatric Ulcerative Colitis Activity Index (PUCAI)-based remission rate was 19.0%. Median partial Mayo score was 6.0 at baseline and 4.0 at Week 30 (overall). Among the eight patients who underwent sigmoidoscopy, Mayo response was achieved at Week 30 (overall) in three patients (37.5%). Trough serum IFX concentrations in Week 8 CAI-based responders were maintained throughout the study period. Adverse events and serious adverse events were observed in 95.2 and 14.3% of patients, respectively.

**Conclusions:**

These results support the use of IFX in the treatment of pediatric patients with UC with inadequate response to existing treatment.

**Trial registration:**

ClinicalTrials.gov, registration number: NCT01585155.

## Background

Ulcerative colitis (UC) is an inflammatory disease of unknown etiology that is characterized by repeating periods of relapse and remission. Patients with UC require long-term medical intervention due to difficulties with treatment. A systematic review reported that the incidence and prevalence of UC vary across different regions around the world and are increasing over time [1]. When age of presentation is considered, 20.0% of patients with UC present with symptoms before the age of 20 [2], and in Japan, 5.9% of patients present with symptoms at ≤16 years [3]. Compared with adult UC, pediatric UC is typically more extensive with a more active disease course [4–7], and requires early treatment augmentation to achieve and maintain disease remission [5, 6].

Patients with moderate-to-severe UC typically receive corticosteroid therapy as primary treatment [5, 6, 8]; however, as corticosteroid therapy affects growth and bone density, it is important to utilize corticosteroid-sparing treatment strategies in pediatric UC [5, 6]. Moreover, corticosteroid dependence is reportedly more common in pediatric versus adult patients with UC. Therefore, administration of appropriate immunomodulators is recommended for those with corticosteroid-dependent disease [4, 6].

Several randomized studies have reported the efficacy and safety of anti-tumor necrosis factor-α agents for adult and pediatric patients with UC [9–12]. Infliximab (IFX), an anti-tumor necrosis factor-α monoclonal antibody, is approved for induction and maintenance treatment of pediatric UC in a number countries outside Japan and is the first biological treatment of choice [6]. In Japan, while IFX is used in pediatric patients based on data from Phase 3 trials in adults with UC [11], the efficacy and safety of IFX have not been demonstrated in a pediatric UC population. Therefore, it is unknown whether the efficacy and tolerability of IFX in Japanese pediatric patients with UC are similar to those in Japanese adult patients.

We conducted the first Phase 3 trial to evaluate the efficacy, safety, and pharmacokinetic (PK) profile of IFX in Japanese pediatric patients with moderate-to-severe UC who have not been adequately treated with current therapy. Specifically, we examined a treatment protocol comprising 5 mg/kg IFX administered at Weeks 0, 2, and 6, with Week 8 responders also receiving treatment at 8-week intervals at Weeks 14 and 22, with a final evaluation at Week 30. This is the first Phase 3 study of IFX in Asian pediatric patients with UC.

## Methods

This multicenter, open-label, uncontrolled, Phase 3 trial was conducted at 17 centers in Japan between April 2012 and September 2014, in accordance with the ethical principles described in the Declaration of Helsinki and in compliance with Good Clinical Practice guidelines. Ethics committee approval was obtained at each participating site. Written informed consent was obtained from each patient’s legal representative and written assent, if possible, from each patient. The trial is registered at ClinicalTrials.gov (NCT01585155; https://clinicaltrials.gov/ct2/show/NCT01585155).

### Patients

Eligible patients were children aged 6–17 years with a diagnosis of UC, a Clinical Activity Index (CAI) score ≥ 7, and a bloody stool score ≥ 2 at screening. All patients were diagnosed with UC ≥3 months prior to the start of the study. Patients also satisfied at least one of the following treatment experiences prior to screening: 1) stable dose of 6-mercaptopurine or azathioprine for ≥4 weeks; 2) stable dose of corticosteroids for ≥2 weeks; 3) inadequate response or adverse drug reaction (ADR) to 6-mercaptopurine or azathioprine in the past 5 years; 4) exacerbation or relapse in response to a reduction in corticosteroid dose or inadequate response to corticosteroids. The main exclusion criteria were patients who had total colitis and were then either found to need a colectomy at registration or satisfied ≥4 of the following criteria: ≥6 episodes/day of bloody diarrhea, intense abdominal pain or rebound tenderness, persistent pyrexia of > 37.5 °C, pulse rate > 90 beats/min, and/or hemoglobin < 8.5 g/dL. Patients who had undergone surgery for UC within 8 weeks of registration, patients who had severe symptomatic fibrotic stenosis in the small or large intestine, and patients with a history of fistulas were also excluded from the study. Patients receiving corticosteroids were to be on stable doses during the study period, and additional usage or dose increase was prohibited, although reducing the dose in an unavoidable medical situation was allowed. Similarly, patients receiving azathioprine, 6-mercaptopurine, or 5-aminosalicylates were to be on stable doses during the study period, and additional usage or dose increase was prohibited, although reducing the dose for reasons other than the primary disease was allowed.

### Study design

**Fig. 1:**
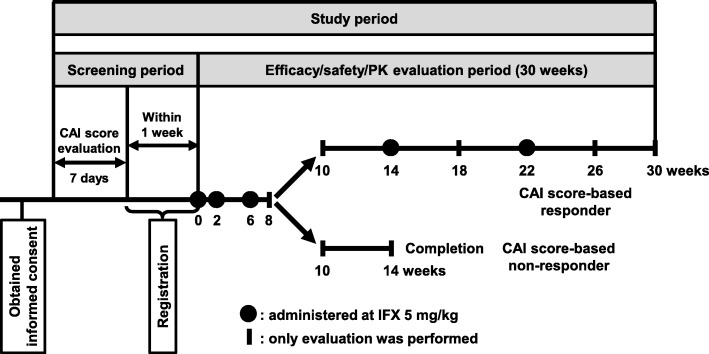
Overview of the study design. *CAI* Clinical Activity Index, *IFX* infliximab. CAI score-based responder: patient who had a decreased (improved) CAI score at Week 8 compared with that measured at the time of registration. CAI score-based non-responder: patient who had an unchanged or increased (worsened) CAI score at Week 8 compared with that measured at the time of registration

**Fig. 2:**
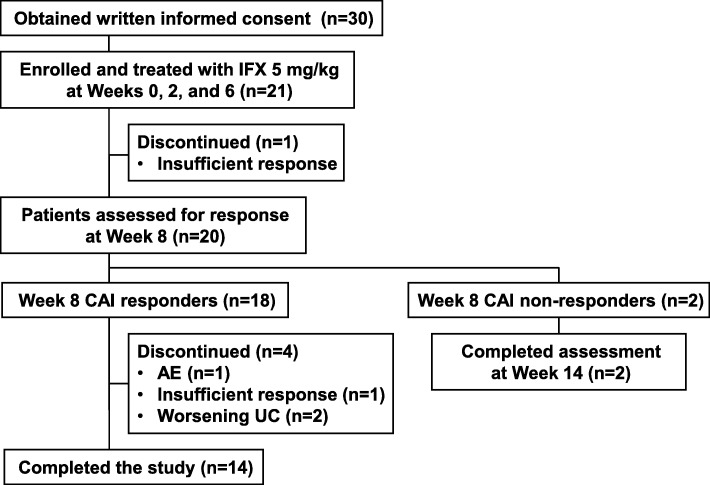
Flow chart of patients throughout the study. *AE* adverse event, *CAI* Clinical Activity Index, *IFX* infliximab, *UC* ulcerative colitis

A summary of the study design and patient flow are presented in Fig. 1 and Fig. 2, respectively. Patients received IFX 5 mg/kg by intravenous infusion over a ≥ 2-h period at Weeks 0, 2, and 6. Patients with a decreased CAI score at Week 8 were defined as responders and received further doses at Weeks 14 and 22. Non-responders (patients with unchanged/increased CAI score) at Week 8 did not receive further treatment (Fig. 2). Assessments were conducted until Week 30 in responders and until Week 14 in non-responders.

### Study endpoints

The study endpoints were efficacy, PK, and safety outcome measures, the results of which were comprehensively evaluated.

#### Efficacy

The efficacy endpoints were change in CAI score, a noninvasive index that is a well-balanced combination of clinical symptoms and laboratory data, and is highly correlated with the Mayo score [13, 14]; percentage of patients who achieved clinical remission (CAI score ≤ 4 [CAI remission]) [15]; Pediatric Ulcerative Colitis Activity Index (PUCAI) score [16]; PUCAI score-based remission (score < 10 at evaluation [PUCAI remission]); and proportion of patients who achieved a PUCAI score decrease of ≥20 points (recommended definition of “response” [16]), measured at the standard evaluation visits at Weeks 0, 2, 6, 8, and 10, and subsequently every 4 weeks until Week 30.

Partial Mayo score (Mayo score [14] without endoscopy) was also measured at the standard evaluation visits, and Mayo score, Mayo score-based response (Mayo score decrease of ≥30% and by ≥3 points and rectal bleeding sub-score decrease of ≥1 point [Mayo response]), Mayo score-based remission (Mayo score ≤ 2 and each of the 4 sub-scores ≤1 [Mayo remission]), and rate of mucosal healing (Mayo sub-score for findings of endoscopy ≤1) were measured at Weeks 0 and 30 in patients who underwent sigmoidoscopy.

Corticosteroid dose, corticosteroid withdrawal rate, and C-reactive protein (CRP) levels were also assessed at the standard evaluation visits.

#### Pharmacokinetics

Serum concentrations of IFX and anti-IFX antibodies (ATI) were assessed at the standard evaluation visits in responders and until Week 14 in non-responders. Concentrations of IFX were measured by enzyme-linked immunosorbent assay using anti-IFX monoclonal antibodies (Janssen Biotech, Inc., Horsham, PA, USA), with a detection limit of 0.10 μg/mL [17]. ATI positivity was also evaluated using enzyme-linked immunosorbent assay [17]. Concentrations of IFX and ATI positivity were measured at Mitsubishi Tanabe Pharma (Osaka, Japan).

#### Safety

Adverse events (AEs) and ADRs were classified according to the Medical Dictionary for Regulatory Activities version 17.1; they were evaluated in responders at Week 8 until Week 30, and in non-responders at Week 8 until Week 14.

### Statistical analyses

As pediatric UC is a rare and relatively intractable disease, the number of pediatric patients with moderate-to-severe disease is small, with an assumed indication of around 1200 patients in Japan when this study was planned, and even fewer patients expected to meet this study’s eligibility criteria. Therefore, a sample size of 20 patients was considered reasonable and feasible. Assuming the Week 8 CAI remission rate in 20 pediatric patients is the same as that in the Japanese Phase 3 trial in adults with UC [11] (38.5% in 78 patients with a CAI score ≥ 7 and a bloody stool score ≥ 2 at the time of registration), the 95% confidence interval (CI) is expected to be 20.7–59.9%. This sample size was deemed sufficient for detecting the occurrence of infections, one of the noted ADRs of IFX therapy, assuming that they occur at the same frequency as in adults.

The efficacy analysis set was defined as the full analysis set that included all patients who received IFX and were evaluated for efficacy at least once. In the event that Week 30 data were missing (due to treatment failure/discontinuation), results from last-observation-carried-forward analysis were included as data at Week 30 and shown as Week 30 (overall). Subgroup analysis according to patient age group (6 to < 12 years and 12 to 17 years) was conducted using Fisher’s exact test. The PK analysis set included those who had received ≥1 dose of IFX and from whom serum IFX concentration or ATI data were obtained at least once after administration of IFX. A subgroup analysis was conducted for trough IFX concentrations among patients who did and did not achieve CAI remission at Week 8 and was conducted as a post-hoc analysis at Week 14. All patients who received IFX treatment with available safety data were included in the safety analysis set.

## Results

### Patients

In total, 30 patients provided informed consent, and 21 were registered and started on IFX treatment. One patient discontinued treatment before the assessment at Week 8 due to insufficient response to IFX. Of the remaining 20 patients assessed at Week 8, 18 were deemed CAI-based responders and received treatment at Weeks 14 and 22. Fourteen of the 18 responders completed the study (Fig. 2). Patients’ characteristics are presented in Table 1.

**Table 1:** Demographics and baseline characteristics (full analysis set)

Characteristic	Number (%) or median (IQR) (total *n* = 21)
Sex	
Male, n (%)	11 (52.4)
Female, n (%)	10 (47.6)
Age (years), median (IQR)	14.0 (12.0, 15.0)
6 to < 12 years, n (%)	4 (19.0)
12 to 17 years, n (%)	17 (81.0)
Weight (kg), median (IQR)	45.40 (40.00, 54.50)
Height (cm), median (IQR)	158.0 (150.0, 162.0)
BMI (kg/m^2^), median (IQR)	17.78 (15.71, 19.55)
Disease duration (years), median (IQR)	2.10 (1.10, 2.80)
Extent of disease	
Limited to left side of colon, n (%)	1 (4.8)
Extensive, n (%)	20 (95.2)
Concomitant medications, at baseline	
Corticosteroids (oral), n (%)	12 (57.1)
Aminosalicylates, n (%)	19 (90.5)
5-aminosalicylates, n (%)	17 (81.0)
Sulfasalazine, n (%)	3 (14.3)
6-mercaptopurine/azathioprine, n (%)	9 (42.9)
Corticosteroid-refractory disease, n (%)	3 (14.3)
Corticosteroid-dependent disease, n (%)	16 (76.2)
CAI score, median (IQR)	9.0 (8.0, 11.0)
Partial Mayo score, median (IQR)	6.0 (5.0, 7.0)
Mayo score,^a^ median (IQR)	7.0 (5.0, 8.5)
PUCAI score, median (IQR)	45.0 (40.0, 60.0)
CRP (mg/dL), median (IQR)	0.20 (0.00, 0.60)
TNF-α (pg/mL), median (IQR)^b^	1.020 (0.000, 1.640)

^a^*n* = 8

^b^Plasma concentration of TNF-α was measured using a chemiluminescence enzyme immunoassay only at baseline because accurate measurement is not possible in the presence of IFX. A measured TNF-α plasma concentration under the detection limit (0.55 pg/mL) was defined as 0.00 pg/mL

*BMI* Body mass index, *CAI* Clinical activity index, *CRP* C-reactive protein, *IQR* Interquartile range, *PUCAI* Pediatric ulcerative colitis activity index, *TNF-α* tumor necrosis factor-α.

### Efficacy

**Fig. 3:**
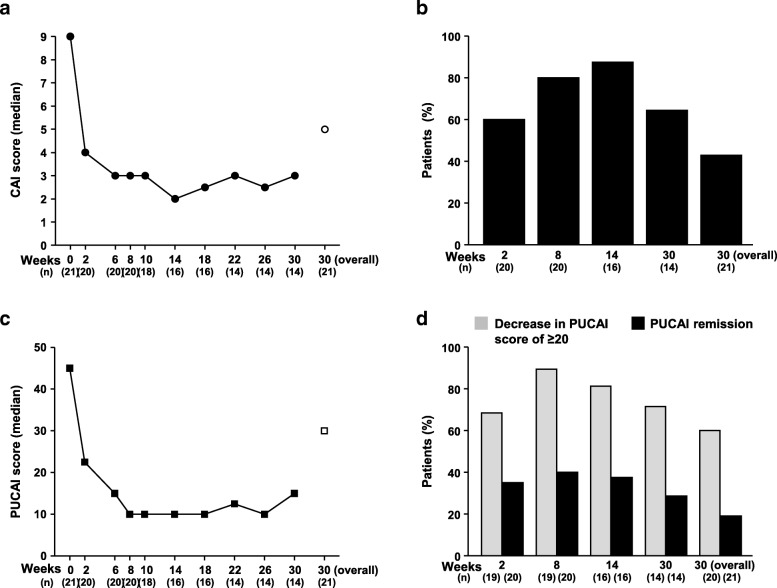
Efficacy responses to IFX treatment over time in Japanese patients with UC. (**a**) Median change in CAI scores. The open circle represents the median CAI score at Week 30 (overall). (**b**) CAI-based remission. (**c**) Median change in PUCAI scores. The open square represents the median PUCAI score at Week 30 (overall). (**d**) PUCAI-based remission and patients who achieved a decrease in PUCAI score of ≥20 over 30 weeks of IFX treatment among patients who achieved a response after 8 weeks of treatment. *CAI* Clinical Activity Index, *IFX* infliximab, *PUCAI* Pediatric Ulcerative Colitis Activity Index, *UC* ulcerative colitis

Marked improvements in the median (interquartile range [IQR]) CAI score were observed from baseline (9.0 [8.0, 11.0]) as early as Week 2 of treatment (4.0 [1.0, 5.5]). The median CAI score at Week 8 was 3.0 (0.5, 3.5), then ranged from 2.0 to 3.0 over Weeks 10 to 30. The median CAI score at Week 30 (overall) was 5.0 (3.0, 7.0) (Fig. 3a). The CAI remission rate at Weeks 2, 8, 14, 30, and 30 (overall) was 60.0% (12/20), 80.0% (16/20), 87.5% (14/16), 64.3% (9/14), and 42.9% (9/21), respectively (Fig. 3b). When missing data were imputed for treatment failure, the CAI remission rate was 57.1% at Week 2, 76.2% at Weeks 6 and 8, and 42.9% at Week 30.

Median (IQR) PUCAI score was 45.0 (40.0, 60.0) at baseline, decreased to 22.5 (2.5, 35.0) at Week 2 and 10.0 (0.0, 22.5) at Week 8, and ranged from 10.0 to 15.0 over Weeks 10 to 30. The median PUCAI score was 30.0 (10.0, 40.0) at Week 30 (overall) (Fig. 3c). Of the patients with a baseline PUCAI score > 20, the percentage who achieved a decrease in PUCAI score of ≥20 was 68.4% (13/19) at Week 2, 89.5% (17/19) at Week 8, 81.3% (13/16) at Week 14, 71.4% (10/14) at Week 30, and 60.0% (12/20) at Week 30 (overall) (Fig. 3d). PUCAI remission rate was 35.0% (7/20) at Week 2, 40.0% (8/20) at Week 8, 37.5% (6/16) at Week 14, 28.6% (4/14) at Week 30, and 19.0% (4/21) at Week 30 (overall).

When missing data were imputed for treatment failure, similar treatment effects were observed on the CAI and PUCAI scores, CAI remission, PUCAI score decrease of ≥20, and PUCAI remission to those obtained without imputations. Subgroup analysis showed no difference in CAI remission rates at Week 8 (100.0% [3/3] and 13/17 [76.5%]) according to age group (6 to < 12 years old and 12 to 17 years old, respectively).

Median (IQR) partial Mayo score was 6.0 (5.0, 7.0) at baseline, decreased to 2.5 (0.5, 4.5) at Week 2, 2.5 (0.0, 3.0) at Week 6, and 1.5 (0.0, 3.0) at Week 8, and then ranged from 1.5 to 3.0 over Weeks 10 to 30. Median (IQR) partial Mayo score was 4.0 (3.0, 4.0) at Week 30 (overall). When accounting for imputed data, the partial Mayo score was 3.0 at Week 2, 3.0 at Week 6, and 2.0 at Week 8, and ranged from 3.0 to 4.0 over Weeks 10 to 30. In the eight patients who underwent sigmoidoscopy, median Mayo score (IQR) was 7.0 (5.0, 8.5) at baseline. Median Mayo score was 5.0 (4.0, 6.0) at Week 30 and Week 30 (overall), and when accounting for imputed data was 4.5 (4.0, 5.5) at Week 30. Mayo response was achieved at Week 30 and Week 30 (overall) in 42.9% (3/7) and 37.5% (3/8) of patients, and Mayo remission was achieved in 14.3% (1/7) and 12.5% (1/8) of patients, respectively.

Mucosal healing was achieved in two out of seven patients at Week 30 (overall) whose endoscopic sub-score was ≥2 at registration.

### Corticosteroid dose

Among patients who were receiving concomitant oral corticosteroids at baseline, median corticosteroid dose (IQR) was 0.20 (0.10, 0.32) (*n* = 12), 0.10 (0.02, 0.16) (*n* = 8), and 0.05 (0.00, 0.16) mg/kg/day (*n* = 12) at baseline, Week 30, and Week 30 (overall), respectively. Median percentage change in the corticosteroid dose was − 1.63, − 25.05%, and − 43.91% at Weeks 2, 6, and 8, respectively, ranged from − 61.72% to − 86.93% over Weeks 10 to 30, and was − 85.44% at Week 30 (overall). When accounting for imputed data, median percentage change in the corticosteroid dose was − 1.63, − 25.05%, and − 43.91% at Weeks 2, 6, and 8, respectively, and ranged from − 45.01% to − 79.31% over Weeks 10 to 30. Following data imputation, the rate of corticosteroid withdrawal was 8.3% at Week 2, 25.0% at Week 8, 25.0% at Week 14, and 16.7% at Week 30. One of two patients who withdrew from corticosteroid use at Week 30 achieved CAI remission.

### CRP measurements

The median (IQR) baseline CRP level was 0.20 mg/dL (0.00, 0.60) and was maintained at 0.00 mg/dL at all time points after Week 2.

### Pharmacokinetics

Overall, median (IQR) IFX concentration 1 h after administration was 97.17 (80.06, 107.43) μg/mL at Week 0 and that before administration was 21.14 (18.91, 24.30) and 10.35 (6.64, 16.55) μg/mL at Weeks 2 and 6, respectively. Median IFX concentration was 25.64 (18.13, 34.72) μg/mL at Week 8. The median (IQR) trough IFX concentration in Week 8 CAI-based responders was 2.58 (0.80, 4.09), 1.54 (0.33, 4.74), and 1.34 (< 0.10, 4.80) μg/mL at Weeks 14, 22, and 30, respectively. In contrast, individual IFX concentrations in Week 8 CAI non-responders (two patients) were 0.26 and 1.74 μg/mL at Week 14. IFX concentration by age group (6 to < 12 years old and 12 to 17 years old) is shown in Table 2.

**Table 2:** IFX concentration by age group

	6 to < 12 years		12 to 17 years	
	μg/mL, median (IQR)	n	μg/mL, median (IQR)	n
1 h after administration at Week 0	92.87 (76.62, 107.71)	4	97.17 (91.53, 107.43)	17
Week 8	20.65 (12.00, 23.08)	3	28.34 (20.39, 35.03)	16
Week 14	0.85 (0.26, 3.45)	3^a^	3.11 (0.80, 6.06)	15^a^
Week 22	0.34 (0.33, 0.57)	3^a^	2.65 (0.48, 5.21)	12^a^
Week 30	1.20 (0.00, 1.48)	3^a^	2.62 (0.00, 5.78)	11^a^

^a^Week 8 CAI-based responders

*CAI* Clinical activity index, *IFX* Infliximab

**Fig. 4:**
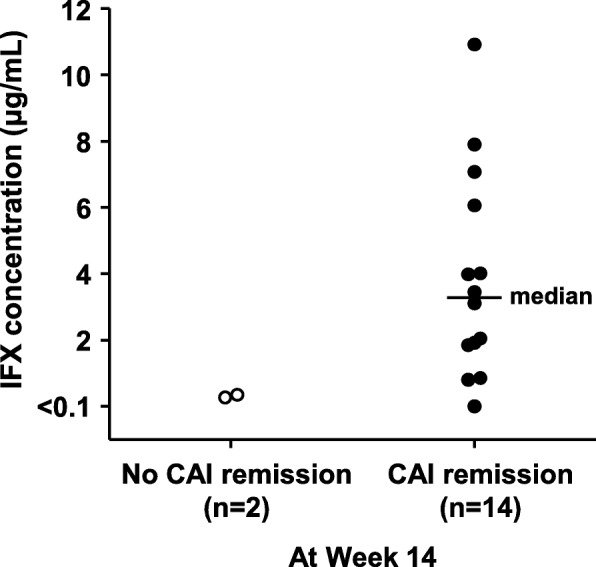
Trough serum IFX concentration at Week 14 in patients who did/did not achieve CAI remission. *CAI* Clinical Activity Index, *IFX* infliximab

Median serum IFX concentrations at Week 8 in patients who did and did not achieve CAI remission among the overall population were 24.42 μg/mL and 26.87 μg/mL, respectively. The median trough serum IFX concentration in patients (*n* = 14) who achieved CAI remission at Week 14 was 3.28 μg/mL, and the individual trough serum IFX concentrations in patients (*n* = 2) who did not achieve CAI remission were 0.26 and 0.35 μg/mL (Fig. 4).

In the overall population, ATI measurements were inconclusive in 81.0% of patients and negative in 19.0% of patients. ATI-negative patients were all responders at Week 8.

### Safety

The incidence rate of AEs and ADRs was 95.2% (20/21) and 71.4% (15/21), respectively (Table 3). The majority of AEs belonged to the system organ class “infections and infestations”, with an incidence rate of 57.1% (12/21). The most common AE was nasopharyngitis (33.3%, 7/21). Serious AEs occurred in 14.3% (3/21) of patients (worsened UC in two patients and enterocolitis in one patient), with 4.8% (1/21, enterocolitis) of patients experiencing serious infection. Worsening of UC in one patient led to the discontinuation of treatment. Similar incidence rates of AEs and ADRs were observed between the two age groups (6 to < 12 years and 12 to 17 years).

**Table 3:** Incidence of AEs

	All *n* = 21	6 to < 12 years *n* = 4	12 to 17 years *n* = 17
Mean duration of follow-up (days)	176.7	169	179
Mean number of infusions	4.3		
AEs, n (%)	20 (95.2)	4 (100.0)	16 (94.1)
ADRs, n (%)	15 (71.4)	3 (75.0)	12 (70.6)
AEs leading to discontinuation of IFX, n (%)	1 (4.8)	0 (0.0)	1 (5.9)
SAEs, n (%)	3 (14.3)	0 (0.0)	3 (17.6)
Serious ADRs, n (%)	1 (4.8)	0 (0.0)	1 (5.9)
Infections, n (%)	13 (61.9)	4 (100)	9 (52.9)
Serious infections, n (%)	1 (4.8)	0 (0.0)	1 (5.9)
Infusion reactions, n (%)	2 (9.5)	0 (0.0)	2 (11.8)
Serious infusion reactions, n (%)	0 (0.0)	0 (0.0)	0 (0.0)

*ADR* Adverse drug reaction, *AE* Adverse event, *IFX* Infliximab, *SAE* Serious adverse event

## Discussion

This is the first Phase 3 study to demonstrate the efficacy and safety of IFX for the treatment of moderate-to-severe UC in Asian pediatric patients. Administration of 5 mg/kg IFX at Weeks 0, 2, and 6 rapidly improved clinical symptoms, and the effects were maintained up to Week 30. IFX also demonstrated a tolerability profile in line with previous investigations.

A Phase 3 study in adult Japanese patients with moderate-to-severe UC showed the usefulness of IFX as induction therapy and maintenance therapy [11]. Among the 78 adult Japanese patients subjected to the same inclusion criteria as the present study (a CAI score ≥ 7 and a bloody stool score ≥ 2 at the time of registration), CAI remission rates (95% CI calculated using the Clopper Pearson method) at Weeks 8 and 30 were 38.5% (27.7–50.2%) and 37.2% (26.5–48.9%), respectively (unpublished data). In the present study, the CAI remission rate (95% CI) was 76.2% (52.8–91.8%) at Week 8 and 42.9% (21.8–66.0%) at Week 30 when missing data were imputed for treatment failure, and were therefore comparable to the rates observed in adult Japanese patients. Almost one-third of patients who underwent sigmoidoscopy achieved mucosal healing in the present study. Despite being unable to directly compare the relative effects of treatment between children and adults, the current results suggest that IFX induces and maintains remission in pediatric patients with UC as effectively as in adult patients with UC.

The present study contained fewer patients aged 6 to < 12 years than those aged 12 to 17 years, making it difficult to conduct a clear comparison between older and younger pediatric patients. However, a subgroup analysis of efficacy results in the current study suggests that IFX treatment was probably as effective in pediatric patients aged 6 to < 12 years as it was in those aged 12 to 17 years, although further investigations are warranted.

A Phase 3 study in non-Japanese pediatric patients with UC demonstrated PUCAI remission rates of 33.3, 40.0, and 38.1% at Weeks 8, 30, and 54, respectively [10]. In the present study, the PUCAI remission rate was similar to that in non-Japanese pediatric patients with UC at Week 8 (40.0%), but was much lower (19.0%) at Week 30 (overall). This difference may be attributed to the increase in dosage in the non-Japanese study from 5 mg/kg to 10 mg/kg at 8-week intervals in approximately 40% of patients before Week 54 due to decreased treatment efficacy, which was defined as “either (1) an increase from the week 8 partial Mayo score of ≥2 points at 2 consecutive visits at least 7 days apart or (2) an increase from the week 8 partial Mayo score of ≥3 points at any visit” [10].

As highlighted earlier, a greater proportion of pediatric patients with UC are corticosteroid-dependent compared with adult patients, and the reduction and withdrawal of corticosteroids is of greater importance for pediatric patients due to their effects on growth and bone density [4–6]. The non-Japanese Phase 3 study in pediatric patients with UC [10] and ACT1 and ACT2 (Active Ulcerative Colitis Trials) in adult patients with UC [12] reported that IFX was effective for the reduction and withdrawal of corticosteroids in these patients. In the present study, among the 12 patients (57.1%) who were using steroids at registration, the dose of corticosteroids decreased after IFX treatment by at most 79.31% up to Week 30, with one of two patients who withdrew from corticosteroid use at Week 30 achieving CAI remission. Although derived from a small number of patients, these results show that IFX administration in pediatric patients with UC is promising for the reduction and withdrawal of corticosteroids, similar to its effects in adult patients with UC.

Serum IFX concentrations during induction therapy and changes in IFX concentrations during maintenance therapy among Week 8 responders in the present study were similar to those reported in the non-Japanese adult Phase 3 study [18]. Data from the present study suggest that the PK of IFX is unlikely to be affected by the age of pediatric patients, similar to that observed in the non-Japanese pediatric Phase 3 study [19].

Previous investigations have reported a relationship between clinical efficacy and serum IFX concentration in adult and pediatric patients with UC [11, 18–21]. In the present study, the efficacy of IFX at Week 8 was not correlated with the IFX serum concentration at this time point, although trough serum IFX concentrations at Week 14 in patients who did not achieve CAI remission tended to be lower than those in patients who achieved CAI remission. We propose that the lack of a correlation between IFX concentration and efficacy at Week 8 may be due to the small number of patients in the study. Larger scale studies are needed to examine the relationship between the trough serum IFX concentration and efficacy in Japanese pediatric patients with UC.

In the present study, the incidence of AEs (95.2%), serious AEs (14.3%), and infections (61.9%) was similar to that observed up to Week 30 in a Japanese Phase 3 study in adult patients (96.2, 17.3, and 59.6%, respectively) [11]. To conduct a detailed investigation of the safety profile of IFX in pediatric patients, we pooled the results of this study and a Japanese Phase 3 study in 14 patients with pediatric Crohn’s disease (CD) [22]. The mean duration of follow-up in this study was 176.7 days compared to 353.9 days in the Japanese Phase 3 study in pediatric patients with CD. There was also a difference in study design, with the Japanese Phase 3 study in pediatric patients with CD including 5 patients who received a dose increase to 10 mg/kg during the study. When the data were pooled, the incidence of AEs, serious AEs, and infections among pediatric patients with inflammatory bowel disease was 97.1% (34/35), 14.3% (5/35), and 65.7% (23/35), respectively, which was similar to that among Japanese Phase 3 adult UC and CD patients [11, 23]. Incidence rates of AEs, serious AEs, and infections in the present study were also similar to those observed in a non-Japanese Phase 3 study in pediatric patients administered 5 mg/kg IFX at 8-week intervals (100.0, 18.2, and 59.1%, respectively) [10], and in ACT1 (87.6, 21.5, and 43.8%, respectively) and ACT2 (81.8, 10.7, and 27.3%, respectively) [12].

The incidence of infections tended to be higher among the younger age group in the present study. However, because no serious events were observed, we speculate that the risk of the infections becoming serious would not be increased in the younger age group. In light of this, the risk of infection among pediatric patients can likely be managed in a similar manner to that in adult patients by using warnings of infection, and no special warnings are needed in the treatment of younger age groups. However, further investigations are warranted due to the small sample size.

Limitations of this study are its small sample size and the lack of a control group. The sample size was decided following consultation with the Pharmaceuticals and Medical Devices Agency based on the rarity of the disease, the target being children with moderate-to-severe disease, and IFX market availability. The small sample size restricted the ability to detect less-common AEs and, ethically, prevented inclusion of a control group. Therefore, the study was examined by comparison with findings from non-Japanese and adult patients.

## Conclusions

For pediatric patients with moderate-to-severe UC with inadequate response to existing treatment, administration of IFX 5 mg/kg at Weeks 0, 2, and 6 rapidly improved clinical symptoms. This improvement was maintained throughout the maintenance treatment period from Week 14 to Week 30 by administration at 8-week intervals. Our results also indicate that IFX treatment was effective for corticosteroid reduction and withdrawal. There were no marked differences in the safety profile compared with approved indications for IFX, confirming the tolerability of this treatment. These results support the use of IFX in the treatment of pediatric patients with UC with inadequate response to existing treatment.

## Data Availability

The datasets generated and/or analyzed in the current study are not publicly available due to ethical reasons (the authors recognize the risk that patients’ identities or private information may be revealed by public data disclosure due to the small sample size and the rarity of the disease studied) but are available from Mitsubishi Tanabe Pharma Corporation (rpp_mtpc@cc.mt-pharma.co.jp) or the corresponding author upon reasonable request.

## Abbreviations

ADR: Adverse drug reaction
AE: Adverse event
ATI: Anti-infliximab antibodies
BMI: Body mass index
CAI: Clinical activity index
CD: Crohn’s disease
CI: Confidence interval
CRP: C-reactive protein
IFX: Infliximab
IQR: Interquartile range
PK: Pharmacokinetic
PUCAI: Pediatric ulcerative colitis activity index
SAE: Serious adverse event
TNF-α: Tumor necrosis factor-α
UC: Ulcerative colitis
